# High contrast two-photon imaging of fingermarks

**DOI:** 10.1038/srep24142

**Published:** 2016-04-07

**Authors:** Caleb R. Stoltzfus, Aleksander Rebane

**Affiliations:** 1 Physics Department, Montana State University, Bozeman MT. 59717, USA; 2 National Institute of Chemical Physics and Biophysics, Tallinn, Estonia, 12618.

## Abstract

Optically-acquired fingermarks are widely used as evidence across law enforcement agencies as well as in the courts of law. A common technique for visualizing latent fingermarks on nonporous surfaces consists of cyanoacrylate fuming of the fingerprint material, followed by impregnation with a fluorescent dye, which under ultra violet (UV) illumination makes the fingermarks visible and thus accessible for digital recording. However, there exist critical circumstances, when the image quality is compromised due to high background scattering, high auto-fluorescence of the substrate material, or other detrimental photo-physical and photo-chemical effects such as light-induced damage to the sample. Here we present a novel near-infrared (NIR), two-photon induced fluorescence imaging modality, which significantly enhances the quality of the fingermark images, especially when obtained from highly reflective and/or scattering surfaces, while at the same time reducing photo-damage to sensitive forensic samples.

Cyanoacrylate fuming is one of the most effective techniques for developing latent fingermarks on non-porous surfaces^1^. After fuming, luminescent stains are often used to visualize the developed fingermarks. When properly applied, luminescent stains permeate the cyanoacrylate deposit with minimal substrate staining. In some key circumstances, such as latent fingermarks developed on aluminum cans or substrates with large background contrast, for example black text on white backgrounds, the fingermark contrast can be compromised due to interference from the substrate. Digital post processing of the images can help to alleviate some of these issues^1^. However, in many cases post-processing is not sufficient, and it is critical to find new ways how to improve raw image quality, especially in challenging circumstances.

UV illumination of stained cyanoacrylate-developed fingermarks is the most common method of imaging this type of fingermark^1^. This method has a few notable drawbacks; there is often high substrate auto fluorescence leading to large background signals and there also exists a risk that UV photons may cause degradation of the specimen^1^,^2^,^3^. Two-photon absorption (2PA) benefits from the ability of some fluorophores, including some organic dyes used in standard forensics, to emit visible fluorescence upon absorbing two NIR photons. This absorption process drastically reduces the detrimental background scattering and background fluorescence, while minimizing specimen degradation. Using this imaging technique, the potential to obtain a high-contrast fingermark is greatly increased.

Two-photon excited fluorescence (2PEF) imaging involves illuminating a sample with a femtosecond laser beam that delivers a high incident photon flux of NIR photons^4^,^5^, sufficient to induce the two-photon transition from the ground state to an excited electronic state of the fluorophore. 2PEF imaging offers intrinsic advantages including; low background scattering, low background fluorescence and high sample photo-stability^6^,^7^,^8^, and has become a standard procedure in biological microscopy. The 2PA process requires that the chromophores possess a large 2PA cross section, σ_2PA_ > 10–10^2 ^GM (1 GM = 10^−50 ^cm^4^ s photon^−1^) at the particular illumination wavelength. Lower background fluorescence occurs due to the highly selective nature of 2PA. Because most naturally occurring fluorophores have a much lower σ_2PA_ value, and also because we can use synthetic stains and dyes where the 2PA maximum lies at NIR wavelengths, the background signal may be largely suppressed. In addition, there is very little or no photo-damage to the sample due to the longer wavelengths and low average illumination powers used.

There are prior reports where two-photon microscopes were used to detect DNA traces, for ultra-sensitive detection of TNT, for the detection of gunshot residue, and for some other applications inside and outside the realm of forensic imaging^2^,^3^,^9^,^10^,^11^,^12^,^13^. To our best knowledge, this is the first report where the distinct advantages of two-photon excitation are implemented in fingermark imaging. Two-photon microscopes have a very limited field of view, typically <1 mm^2^. Forensic fingerprint imaging requires a field of view of at least a few cm^2^. We have recently developed a set of new two-photon illumination and imaging tools that have a 10 cm^2^, or larger, field of view^5^,^14^, i.e. sufficient for imaging objects such as beverage cans, door knobs, computer equipment etc., where most forensically significant fingermarks tend to occur. Here, we describe the use of these tools for 2PEF imaging of latent fingermarks on standard aluminum cans, even though a broad range of other common substrates may also be applied.

The technical details of our wide field of view 2PEF imaging system, shown in Fig. 1, have been described in detail elsewhere^5^,^14^. Briefly; a Ti:Sapphire regenerative amplifier system (Coherent Legend-HE) pumped by a 1 kHz Nd:YLF laser (Coherent Evolution) is used for two-photon excitation. After pulse compression, the amplifier output has a peak wavelength of 790 nm, pulse duration of 150 fs and pulse energy of 1.3 mJ. A reference photodiode (PD) is used to monitor the average laser power. The excitation laser beam is scanned in the vertical and horizontal directions using an x-y-axis motorized mirror mount (SM) (Zaber T-OMG). For scanning convenience the excitation laser beam is formed into a stripe using the combination of a cylindrical lens (L1) and a spherical lens (L2). The fluorescence signal is detected with a TE-cooled CCD camera (Hamamatsu C4742-98). The 2PEF is measured by scanning the laser illumination across the sample while integrating the fluorescence on the camera. A LabVIEW program is used to control and acquire data from the CCD camera and to control the scanning mirror. The 2PEF image is recorded by averaging 40 acquisitions, with an 8 second exposure time per acquisition. During each acquisition, the laser focus is raster-scanned over the entire field of view of the sample, by moving the beam continuously in the y-direction, and in half beam width steps in the x-direction. A stack of color filters are used in front of the CCD camera (F1) to select the desired fluorescence emission wavelength and block any scattered excitation light. To acquire the linear fluorescence images, the laser beam is blocked, and the sample is illuminated with 395 nm light from a stack of 9 UV LEDs, with the total power ~30 mW and average power density at the sample, 0.3 mW cm^−2^. The UV image is acquired by averaging 4 acquisitions, with a 2 second exposure time per acquisition.

To test the efficacy of 2PEF imaging of stained cyanoacrylate-developed fingermarks, fingermarks were deposited on the bar code of an aluminum can. After depositing the fingermarks, the aluminum can was immediately placed in a cyanoacrylate fumigation chamber. Super glue was evaporated in the camber and the aluminum can was allowed to sit for 10 minutes. Once removed from the chamber, the aluminum can was checked to ensure that the fingermark had been fully developed. The cyanoacrylate deposit was then allowed to dry for at least 24 hours. Once the cyanoacrylate deposit was fully dry it was washed with a solution of Rhodamine 6G dissolved in Methanol. The aluminum can was then imaged with the system described above. The same fluorescence detection filters were used for both the 2PEF imaging and the UV illumination.

Figure 2 shows two raw images of the same fluorescent fingermark taken with 2PEF imaging and UV illumination. The yellow line shows the pixel intensity along a cross section of the image (black line). Under UV illumination (Fig. 2b) the image of the fingermark has sufficient contrast in the region of the image with a white background (right part of image), but little to no contrast in the region of the image with a black background (left part of image). The lack of contrast on the left part of image makes analysis of the complete fingermark difficult. The 2PEF image of the aluminum can (Fig. 2a) shows sufficient contrast on both the white, and black parts of the aluminum can.

The yellow line in Fig. 2 highlights the quality of the 2PEF image of the fluorescent fingermark and demonstrates that this alternative illumination technique is almost completely independent of the substrate. Figure 3 shows the relative pixel count of the UV and 2PEF signal taken from the dark background (a) and bright background (b) regions. To present a more quantitative measure of the image quality, we determine the average fingermark contrast at 66 different locations on 4 different fingermark images (see Supplementary Information), including both bright- and dark backgrounds, as shown by the bar charts in Fig. 4. The contrast was determined by subtracting minimum pixel values from the adjacent maximum values, and averaging over a small area. By defining the contrast in this manner, we are able to assess how well a fingermark can be identified either on a bright or dark background. On average, the 2PEF images show at least a factor of two higher contrast than the corresponding UV images.

We have shown that femtosecond NIR excitation of two-photon induced fluorescence, in conjunction with the selective nature of the two-photon absorption of a common dye, Rhodamine 6G, greatly enhances the image contrast of fingermarks on a reflecting surface. Thus offering a distinct advantage over conventional UV-illumination, especially when dealing with high contrast backgrounds like aluminum cans. The 2PEF images of fingermarks have, on average, a factor of two higher contrast compared to the images obtained with the standard UV illumination technique. 2PEF images show negligible detrimental effects from the substrate reflectivity/scattering or substrate fluorescence. The use of two-photon dyes, like TVP2, could further enhance the benefits of two-photon imaging.

## Additional Information

**How to cite this article**: Stoltzfus, C. R. and Rebane, A. High contrast two-photon imaging of fingermarks. *Sci. Rep*. **6**, 24142; doi: 10.1038/srep24142 (2016).

## Supplementary Material

Supplementary Information

## Figures and Tables

**Figure 1:**
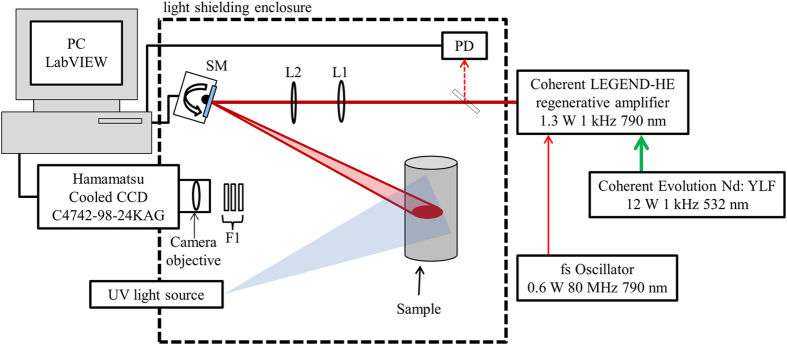
Schematic of the 2PEF imaging setup. L1, cylindrical lens; L2, spherical lens; PD, photo diode; SM, scanning mirror; F1, fluorescence detection filters.

**Figure 2:**
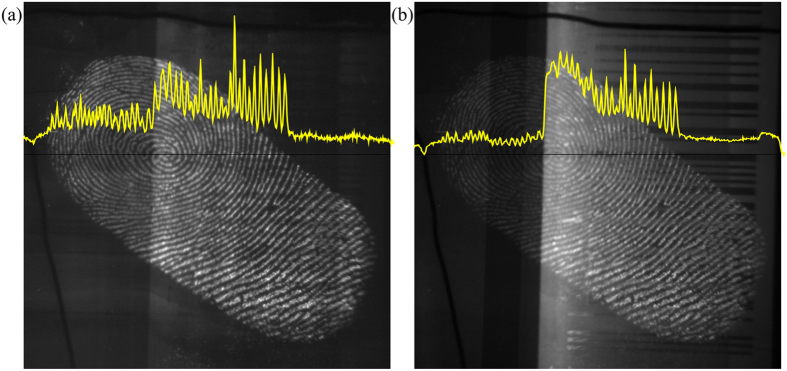
Aluminum can with a stained latent fingermark developed using cyanoacrylate fuming. (**a**) Image of the stained fingermark using 2PEF imaging. (**b**) Image of the stained fingermark using UV illumination. The yellow line shows the pixel intensity along a cross section of the image (black line). More fingermark images are shown in the Supplementary Information.

**Figure 3:**
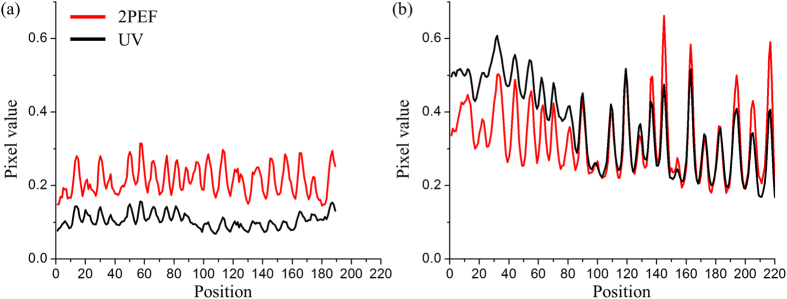
The pixel values along the cross section of the image shown in Fig. 2. (**a**) The left half of the image with a black background. (**b**) The right half of the image with a white background. The red line represents the pixel values from the 2PEF image. The black line represents the pixel values from the UV image.

**Figure 4:**
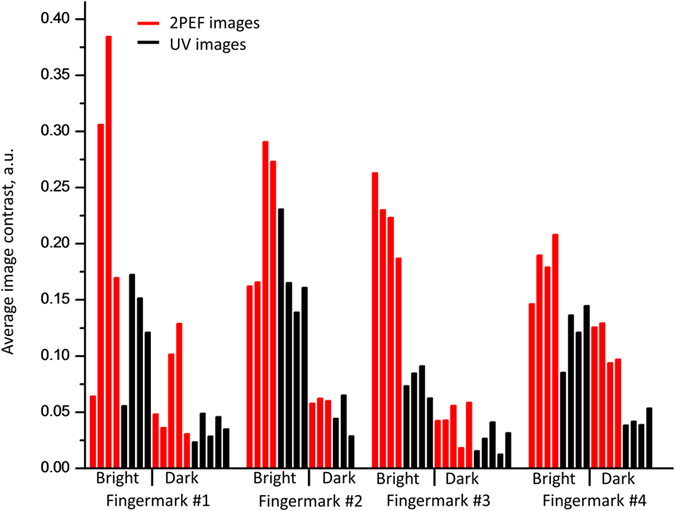
Average contrast of fingermarks depending on the mode of illumination. A column represents one selected region of either 2PEF (red) or UV (black) image (see Supplementary Information).
