# Supplementary material for: High contrast two-photon imaging of fingermarks
Source: Sci Rep. 2016 Apr 7;6:24142. doi: 10.1038/srep24142 (PMC4823789; doi:10.1038/srep24142)
Supplement: Supplementary Information [file srep24142-s1.doc]

High contrast two-photon imaging of fingermarks

Caleb R. Stoltzfus^a^, Aleksander Rebane^a,b^,*

^a^Physics Department, Montana State University, Bozeman MT. 59717; ^b^National Institute of Chemical Physics and Biophysics, Tallinn, Estonia, 12618, *Corresponding author

**Supplementary Information**


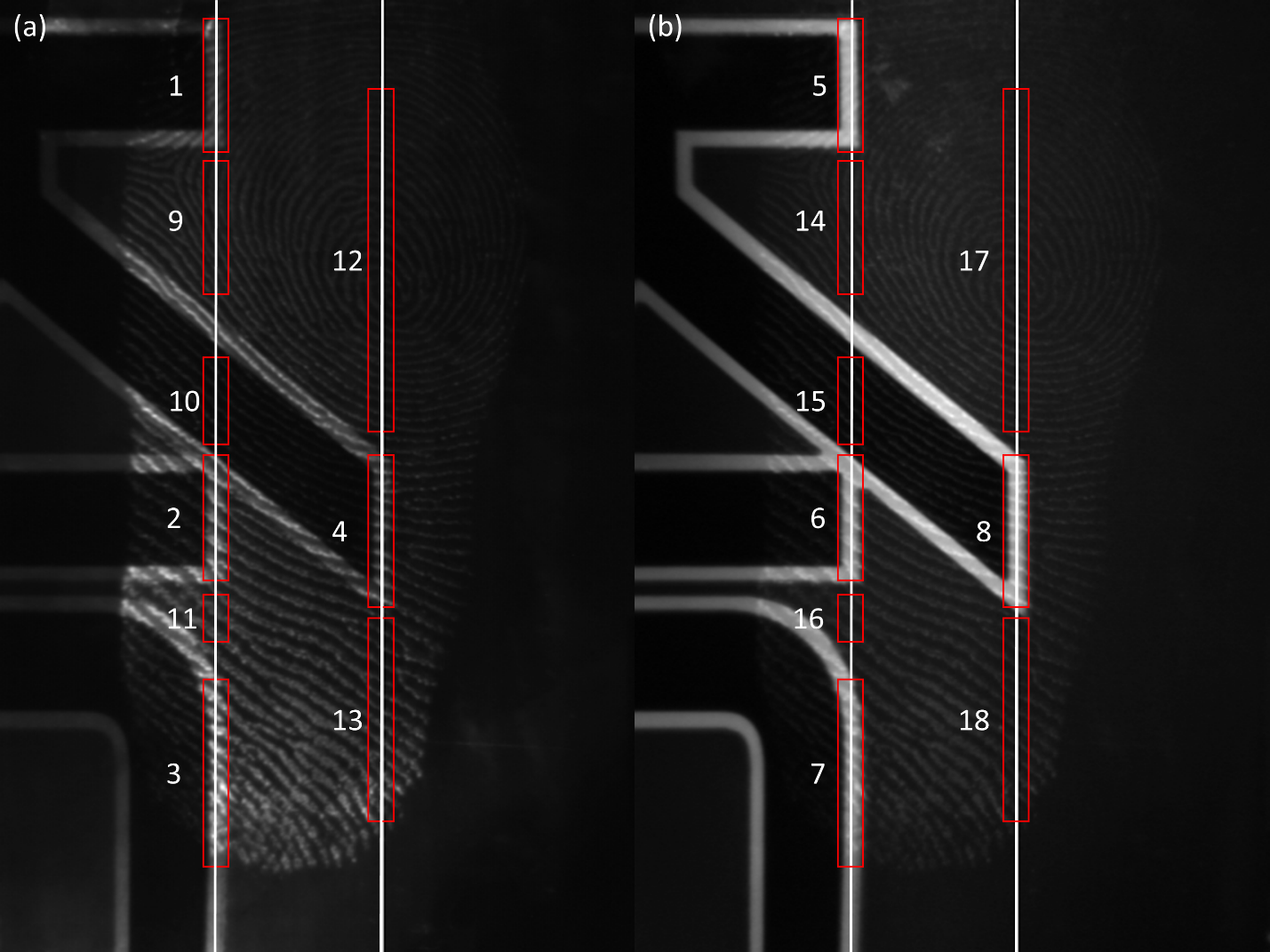


**Supplementary Figure 1| Fluorescent images of fingermark number 1.** (a) Image of the stained fingermark using 2PEF imaging. (b) Image of the stained fingermark using UV illumination. The white lines highlight the rows of pixels for which the contrast was calculated. The contrast was calculated for each numbered region and is shown in Supplementary Figure 5.


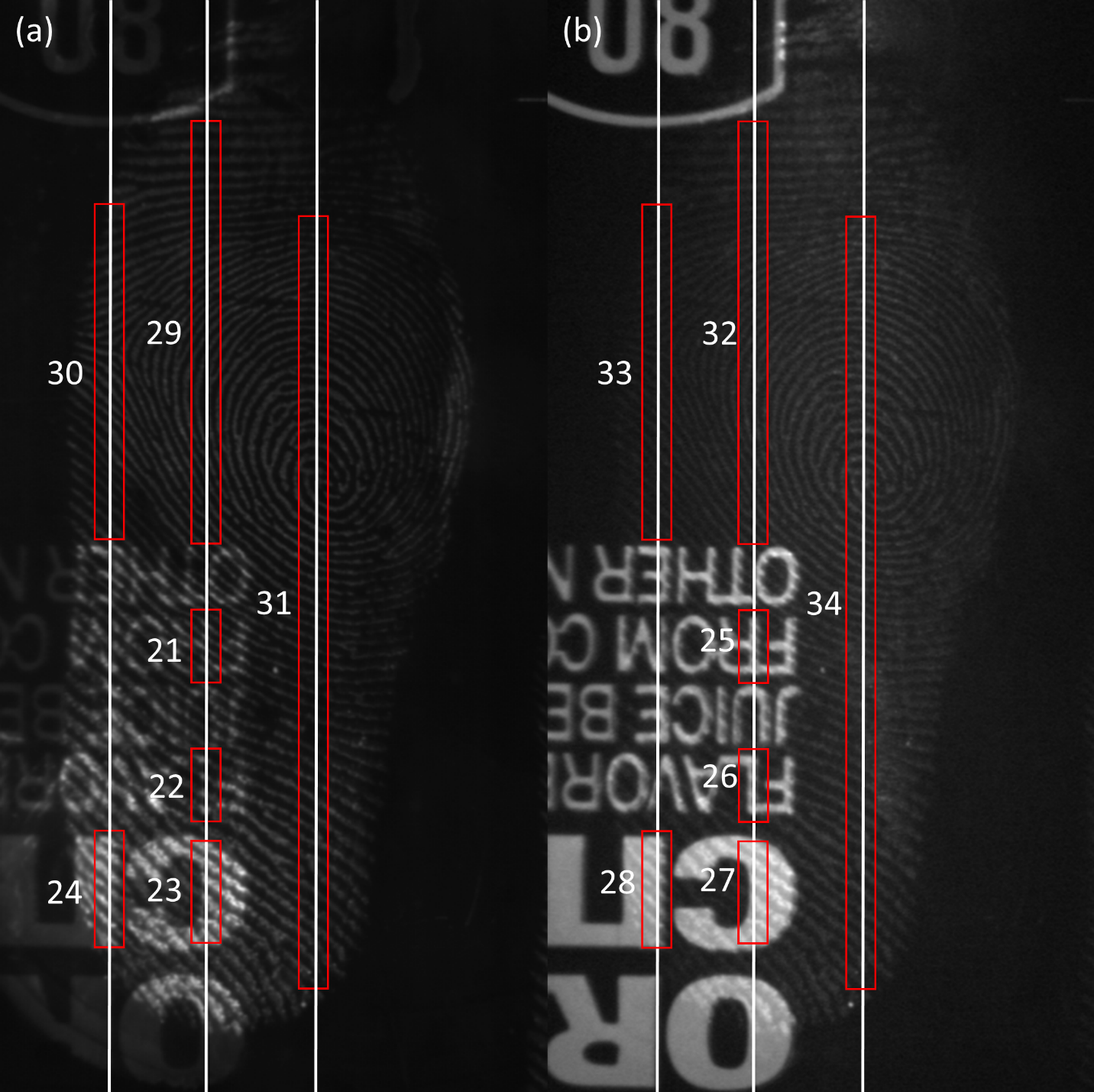


**Supplementary Figure 2| Fluorescent images of fingermark number 2.** (a) Image of the stained fingermark using 2PEF imaging. (b) Image of the stained fingermark using UV illumination. The white lines highlight the rows of pixels for which the contrast was calculated. The contrast was calculated for each numbered region and is shown in Supplementary Figure 5.


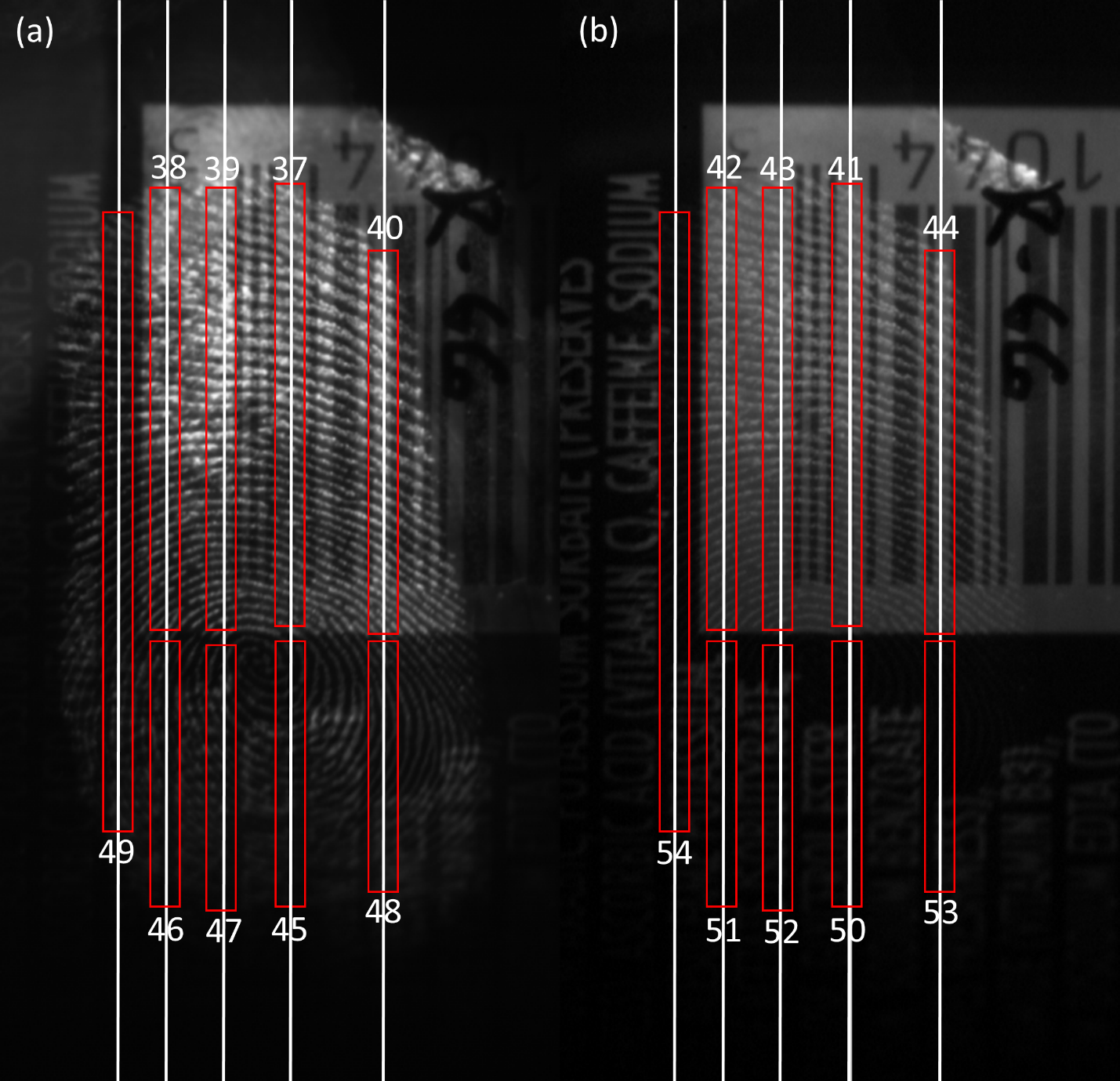


**Supplementary Figure 3| Fluorescent images of fingermark number 3.** (a) Image of the stained fingermark using 2PEF imaging. (b) Image of the stained fingermark using UV illumination. The white lines highlight the rows of pixels for which the contrast was calculated. The contrast was calculated for each numbered region and is shown in Supplementary Figure 5.


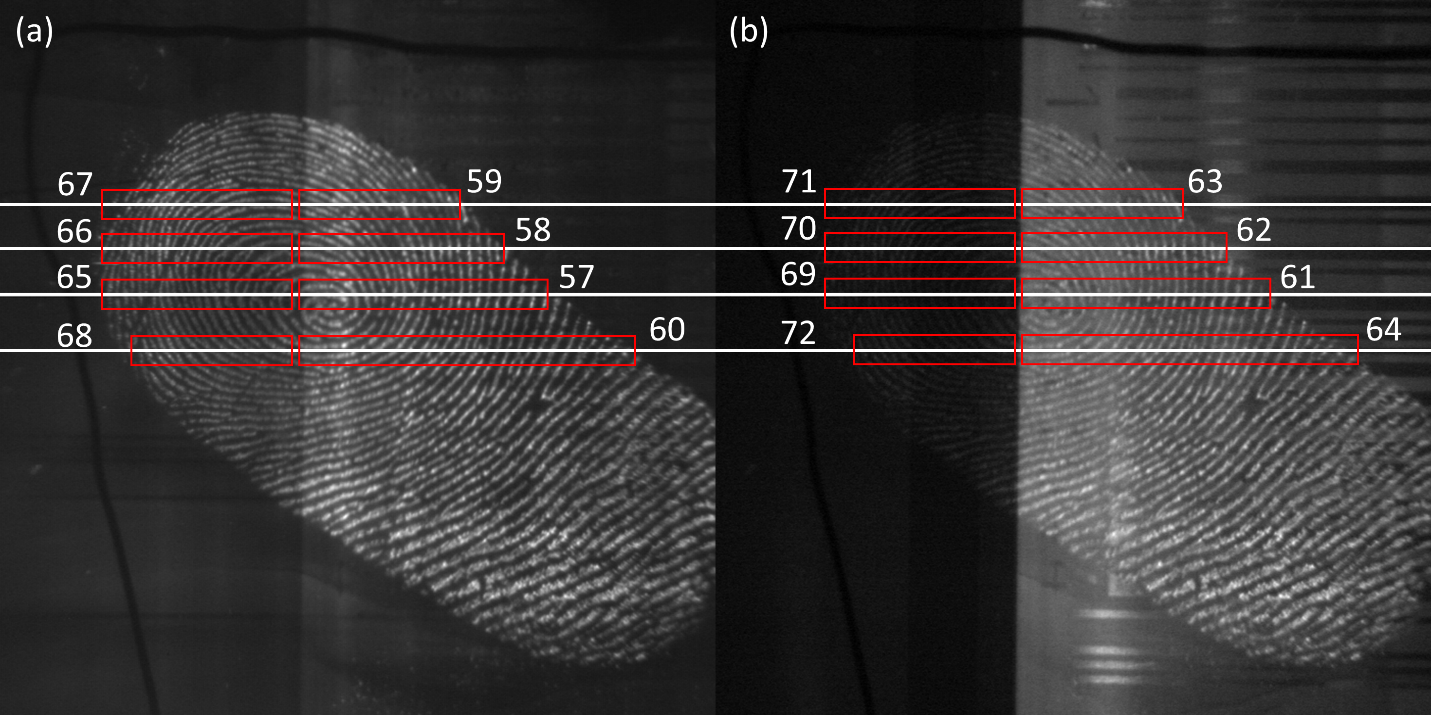


**Supplementary Figure 4| Fluorescent images of fingermark number 4.** (a) Image of the stained fingermark using 2PEF imaging. (b) Image of the stained fingermark using UV illumination. The white lines highlight the rows of pixels for which the contrast was calculated. The contrast was calculated for each numbered region and is shown in Supplementary Figure 5.


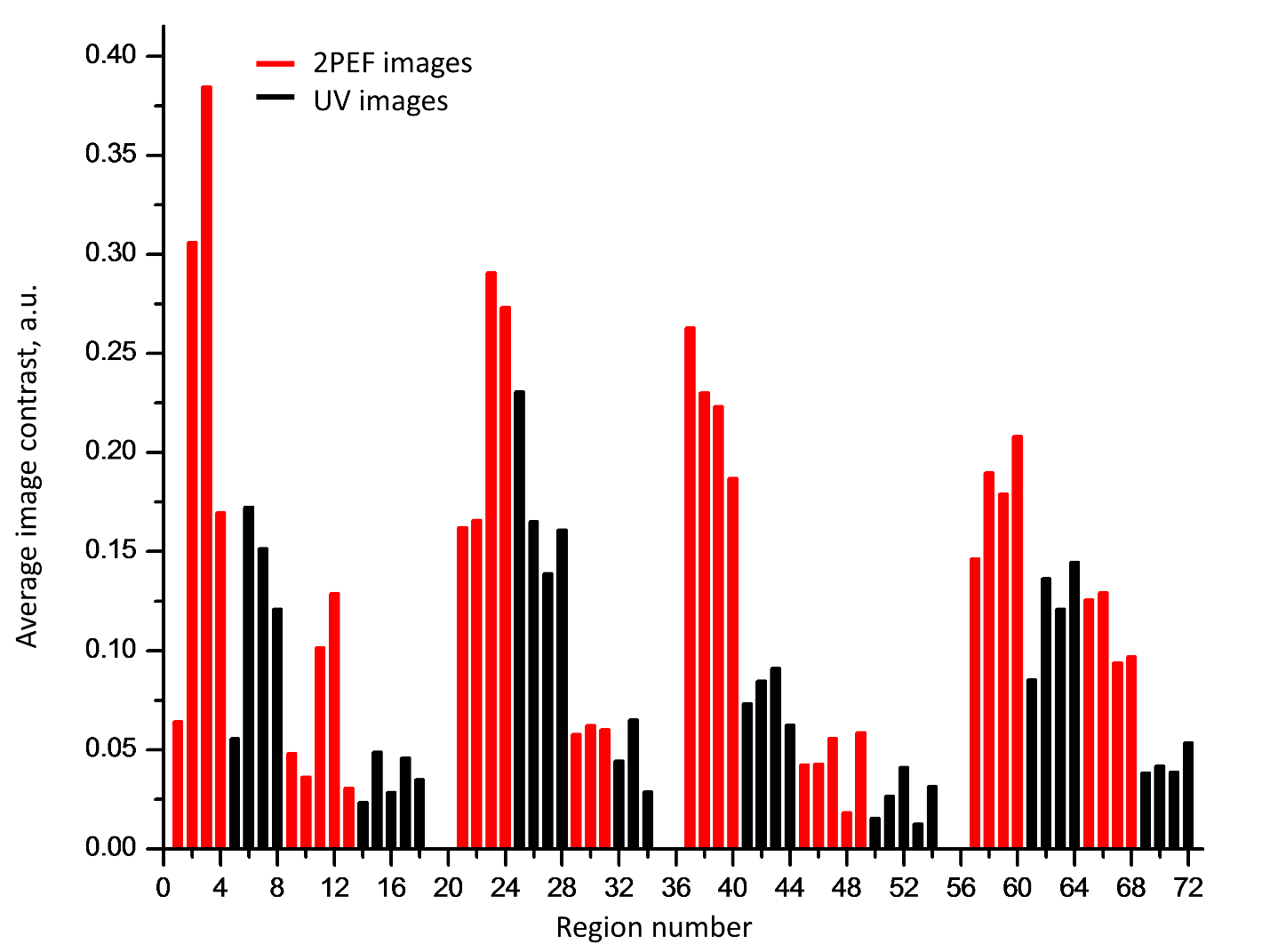


**Supplementary Figure 5| Contrast histogram.** This plot shows the value of the average contrast of the different regions from the fingermark images shown above.
